# Spatially localized sono-photoacoutic activation of phase-change contrast agents

**DOI:** 10.1016/j.pacs.2020.100202

**Published:** 2020-08-03

**Authors:** David S. Li, Geng-Shi Jeng, John J. Pitre, MinWoo Kim, Lilo D. Pozzo, Matthew O’Donnell

**Affiliations:** a Department of Bioengineering, University of Washington, Seattle, WA, 98195 USA; b Department of Chemical Engineering, University of Washington, Seattle, WA, 98195 USA

**Keywords:** Photoacoustics, Ultrasound, Sono-photoacoustics, Phase-change contrast agents, Nanodroplets, Theranostics

## Abstract

Sono-photoacoustic (SPA) activation lowers the threshold of phase-change contrast agents by timing a laser shot to coincide with the arrival of an acoustic wave at a region of interest. The combination of photothermal heating from optical absorption and negative pressure from the acoustic wave greatly reduces the droplet’s combined vaporization threshold compared to using laser energy or acoustic energy alone. In previous studies, SPA imaging used a broadly illuminated optical pulse combined with plane wave acoustic pulses transmitted from a linear ultrasound array. Acoustic plane waves cover a wide lateral field of view, enabling direct visualization of the contrast agent distribution. In contrast, we demonstrate here that localized SPA activation is possible using electronically steered/focused ultrasound pulses. The focused SPA activation region is defined axially by the number of cycles in the acoustic pulse and laterally by the acoustic beam width. By reducing the spot size and enabling rapid electronic steering, complex activation patterns are possible, which may be particularly useful in therapeutic applications.

## 1 Introduction

Phase-change contrast agents are a new class of medical ultrasound (US) imaging agent emerging as an alternative to conventional gaseous perfluorocarbon (PFC) microbubbles [[1], [2], [3], [4], [5]]. They are liquid droplets with a PFC core stabilized with a lipid, protein, polymer, or particle-based shell [[6], [7], [8], [9], [10]]. Although they can be synthesized using core and shell materials chemically identical to currently FDA approved gaseous microbubble contrast agents, they differ from microbubbles in that they are stored and delivered as liquid droplets [[10], [11], [12]]. In liquid state, they are acoustically transparent, preventing US shadowing. Additionally, they are stable and can be stored for as long as weeks to months [10,12,13].

For US imaging, phase-change contrast agents must be vaporized to form microbubbles. Because the molar density of PFCs are much greater in liquid versus gaseous state, the resulting bubbles are approximately 125 times volumetrically larger (5 times diametrically) than the initial droplet [[14], [15], [16], [17], [18]]. This means that nanodroplets under 200 nm in diameter can vaporize to form microbubbles of similar size to current FDA-approved microbubbles. While conventional ultrasound microbubbles have a mean diameter on the order of 1 μm, restricting them to the vasculature, nanodroplets with diameters under 200 nm can freely diffuse past blood vessel walls (i.e., extravasate) via the enhanced permeability and retention (EPR) effect [[19], [20], [21]]. Moreover, in the liquid nanodroplet phase, the agents are acoustically transparent, providing little ultrasound contrast.

After activation the resulting bubbles are micron-scale PFC microbubbles that provide excellent ultrasound contrast. Depending on size, interfacial properties, and PFC volatility, the resulting microbubble will either remain a stable gas bubble or recondense back into a liquid droplet, which can be repeatedly reactivated [22,23]. Furthermore, because of their small diameters, several studies have shown a significant increase in nanodroplet stability due to increased contributions from interfacial tension, enabling perfluorcarbon droplets with bulk boiling points as low as −37 °C [24]. This greatly increases shelf life (stable for weeks to months) and also suppresses spontaneous vaporization of low boiling point PFC droplets [10,12,24].

Given all of their advantages, however, nanodroplets have not rapidly replaced microbubbles for one primary reason. The activation threshold significantly rises with decreasing droplet diameter [13,18,25,26]. In some cases, the threshold is prohibitively high, greatly exceeding acoustic FDA limits for acoustic excitation and optical ANSI safety limits for optical excitation. Although volatile gaseous PFCs, as opposed to liquid PFCs, have reduced activation thresholds, they are challenging to handle and it is difficult to avoid spontaneous droplet vaporization. Alternatively, modifying the activation method to promote vaporization at lower thresholds is possible by combining multiple energy sources.

Sono-photoacoustics (SPA) is a non-linear droplet vaporization method combining acoustic and laser pulses [27,28]. It relies on photothermal heating at the interface of a droplet coated with an optical absorber coincident with the rarefaction phase of an acoustic wave to greatly lower the activation threshold. SPA has been shown to lower the threshold by at least an order of magnitude relative to optical or acoustic activation alone (optical fluences of <1 mJ/cm^2^ and acoustic negative pressures <1 MPa using SPA, versus ∼100 mJ/cm^2^ or ∼5 MPa using optical or acoustic activation alone) [27,29]. Under most clinical imaging conditions, light entering the body is rapidly diffused with depth due to strong optical scattering. Consequently, photoacoustic (PA) imaging depth is often limited to a few centimeters at most. However, with SPA imaging, nanodroplet activation can still be achieved by modulating the acoustic pressure relative to the optical pulse energy at depths impossible for conventional optical activation [27,29]. Similarly, modulating the acoustic pressure relative to the optical fluence can increase the contrast-to-noise ratio (CNR) of SPA imaging [27,29].

**Fig. 1:**
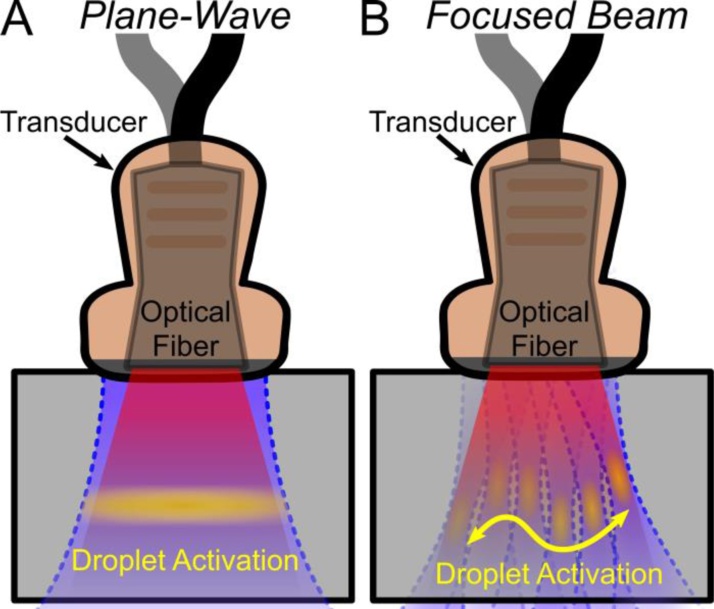
Illustration showing broad beam SPA droplet activation using (A) plane-wave ultrasound versus localized activation using (B) steered and focused ultrasound pulses. Assuming droplets are evenly distributed in the media and light broadly illuminates the imaging domain, the dimensions of the SPA activation volume are determined by the lateral acoustic beam width and the N-cycles used during ultrasound transmission.

Previous studies on SPA imaging transmitted an unfocused acoustic plane wave followed by a delayed laser pulse arriving coincident with the acoustic wave at the region of interest [[27], [28], [29]]. Plane-waves activate a wide lateral width, nearly ideal for high frame rate imaging (Fig. 1A). In this study, we demonstrate that a steered and focused acoustic beam can perform highly localized activation (Fig. 1B) that is more appropriate for a number of applications. For example, at acoustic intensities well above the threshold, nanodroplets are fully vaporized and resultant bubbles violently cavitate to ablate tissue [[30], [31], [32]]. In theory, droplet activation can be as small as the acoustic beam width in the lateral direction and a half wavelength along the axial direction. Therefore, precise activation is more appropriate than broad-beam activation for targeted ablative therapies using SPA, where raster scanning a treatment area and minimizing collateral damage is required.

## 2 Materials and methods

### 2.1 Agent synthesis

Polypyrrole (PPy) coated PFC droplets were synthesized using methods described by Li et al. [29] In summary, a base stock of PFC nanodroplets were spontaneously nucleated using the ouzo method [12]. Polyvinyl alcohol (PVA) dissolved in water was added to stabilize the newly nucleated PFC droplets to achieve a concentration of 1 wt. %. A dissolved solution of iron (III) chloride was added to the PVA coated droplet solution at a molar iron to PVA chain ratio of 200:1. The iron was stirred at 4 °C for 20 min to form iron-PVA complexes. Finally, pyrrole monomer was added for an iron to pyrrole molar ratio of 2:1 and stirred overnight at 4 °C to polymerize, forming a PPy shell around the droplets. An excess of iron was used to ensure complete polymerization of all free pyrrole monomers.

Once the polymer was fully polymerized, samples were centrifuged and resuspended in water three times to remove excess alcohol and free PPy particles that may have polymerized. The droplets had a broad absorption spectrum covering the visible and near-infrared spectrum, with a peak near 1064 nm (see supplemental Fig. 1 for extinction spectrum. Perfluoropentane (T_Boiling_ = 29 °C) was used as the core PFC for droplets that recondense after activation, while perfluorobutane (T_Boiling_ = −2 °C) was used for irreversible activation. The average droplet diameter for the PPy coated perfluoropentane droplets were 186.2 nm (standard deviation = 44.2 nm), while the PPy coated perfluorobutane droplets were 216.7 nm (standard deviation = 40.0 nm) (See supplemental Fig. 2 for size distributions).

### 2.2 Phantom synthesis

PVA gel phantoms embedded with PPy coated droplets were synthesized with methods adapted from Hyon et al. [33] Titanium dioxide nanoparticles were dispersed using sonication, at a concentration of 0.001 % by mass, into a mixture of dimethylsulfoxide (DMSO) and water at a 4:1 vol ratio. The resulting DMSO and water solution dispersed with titanium dioxide nanoparticles was then heated to a minimum of 110 °C on a stir plate as PVA was added to the solution to achieve a final concentration of 2% by weight. After the PVA was fully dissolved, the solution was degassed using a vacuum chamber and cooled to 35 °C before adding PPy coated droplets to achieve a concentration of approximately 1 × 10^6^ droplets/mL of phantom gel. The droplet laden PVA gel was then cast into gel blocks (5.5 cm by 5.5 cm by 4 cm) and allowed to gel at -20 °C for 12 h. The PVA gel phantoms were then stored at 20 °C in a DI water bath. The DI water was regularly exchanged every 12 h with fresh water to remove DMSO from the phantoms for a minimum of 48 h prior to use.

Titanium dioxide nanoparticles were included in the phantoms to mimic in vivo optical conditions, diffusing entering light throughout the imaging volume. Transmission through the phantom material was measured using UV–vis spectrophotometry to estimate local laser fluences. The effective optical attenuation coefficient in the phantom was 0.88 cm^−1^ at the 1064 nm optical wavelength used for the SPA measurements described below.

### 2.3 Ultrasound measurements

A Verasonics Vantage 128 (Verasonics Inc., Kirkland, WA, USA) paired with a linear array ultrasound transducer (ATL L7−4) was used for imaging and droplet activation. The linear array featured 128 elements (0.298 mm element pitch) with a lateral aperture of 38 mm, elevational aperture of 7 mm, and a fixed elevational focus at 25 mm. The beam width in the elevational direction at the elevational focus was measured to be 3.1 mm using a needle hydrophone (HN-1000, Onda Corp., Sunnyvale, CA, USA). The center frequency was 5.2 MHz with -6 dB frequency response at 3 MHz–7.2 MHz. All experiments were performed within the depth of field of the elevational lens. The acoustic beam profile was primarily determined by the f-number (where f = focal length/acoustic aperture dimension) of the lateral aperture of the active array elements. Optical pulses (5 ns duration) at 1064 nm wavelength at 20 Hz from an Nd:YAG laser source (Surelite SL I-20, Amplitude Lasers, Continuum, CA, USA) were fiber coupled to a custom bifurcating fiber bundle producing a 1 mm by 15 mm diverging beam (NA = 0.2) coaxially aligned to the direction of sound propagation and angled such that the illumination field overlapped with the elevational focus of the linear ultrasound array. A block diagram and photos of the transducer and optical fiber arrangement can be found in supplemental Fig. 3. The laser was calibrated using a calibrated energy meter (Nova II, Ophir-Spiricon LLC, North Logan UT, USA). In all studies, the ultrasound probe and optical fibers were positioned at the top of the imaging frame firing downward along the axial direction. Laser pulses were triggered by the Verasonics system such that the laser flash coincided with the arrival of a focused ultrasound pulse at the region of interest. Imaging was performed in a degassed water tank at room temperature.

**Fig. 2:**
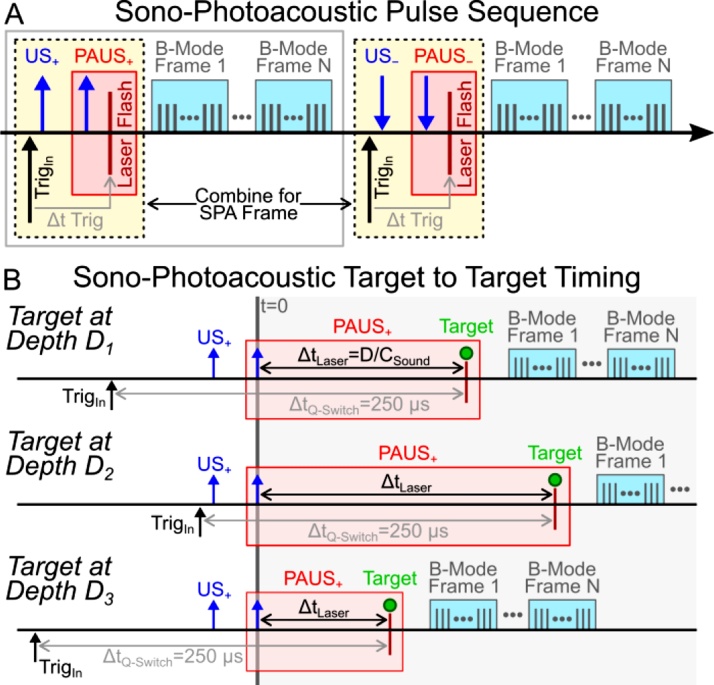
(A) SPA timing diagram for a single frame. One SPA frame requires four ultrasound shots, two of which are synchronized with a laser shot such that the laser pulse arrives with the acoustic wave at the target location. Between SPA subframes, B-mode ultrasound image acquisitions are interleaved. (B) A more detailed view highlighting the SPA activation timing delays (zooming in on gray boarded box in panel A) of the acoustic activation pulses relative to the laser flash from frame to frame. From shot to shot, the SPA activation target depth (represented by the green circle) can vary (e.g., D_2_>D_3_>D_1_). For the laser flash to coincide with the arrival of the acoustic pulse at the target position, the laser flash delay (Δt_Laser_) must shift relative to the first element firing from the transducer (t = 0 μs). However, to stabilize the laser energy output, the laser trigger frequency (20 Hz) and Q-switch delay (Δt_Q-Switch_) must remain constant. As a result, from an absolute global timing perspective, the laser trigger frequency is constant while the SPA subframe firings adjust according to time delays necessary to perform SPA activation at a variable depth from frame to frame.

The SPA image sequence requires 4 subframes to remove all linear PA sources and acoustic scatters (Fig. 2A). The basic sequence is described in detail by Arnal et al. [28]. In summary, linear signal cancellation isolates non-linear droplet activation using the relation: $SPA=\left(PAUS_{+}-US_{+}\right)+\left(US_{-}-PAUS_{-}\right)$. US refers to a focused ultrasound pulse without a laser pulse while PAUS pulses refer to identical ultrasound pulses with a delayed laser pulse such that the laser flash coincides with the arrival of the focused ultrasound pulse. The +/- subscripts refer to positive or negative phases of transmitted acoustic pulses.

Because the laser firing rate was limited to 20 Hz, real time SPA images were produced at a 10 Hz frame rate. With the 50 ms delay between firings, two full B-mode frames containing 64 A-lines per frame were acquired between laser firings (Fig. 2A) and displayed at 40 Hz. A function generator produced a 20 Hz trigger to the flashlamp of the 1064 nm laser as well as initiate the Verasonics sequence. However, the Verasonics produced the final trigger, flashing the laser 250 μs after the initial trigger. The Verasonics system acted as the master timing control to minimize PA and PAUS signal jitter. Time delays for US, PAUS, and B-mode frames were precomputed based on activation path distances and shifted relative to the laser firing (Fig. 2B) to obtain steady 20 Hz laser triggering (10 Hz SPA) and an average 40 Hz B-mode ultrasound frame rate. The number of active elements used for SPA activation were dynamically adjusted shot to shot depending on the activation location to maintain a constant f-number. All images were normalized according to the maximum signal acquired during acquisition and displayed on a log-compressed scale. SPA contrast images, displayed using a hot colormap, were superimposed on the ultrasound images, displayed using a grayscale colormap.

## 3 Results and discussion

### 3.1 SPA activation at variable depth and position

**Fig. 3:**
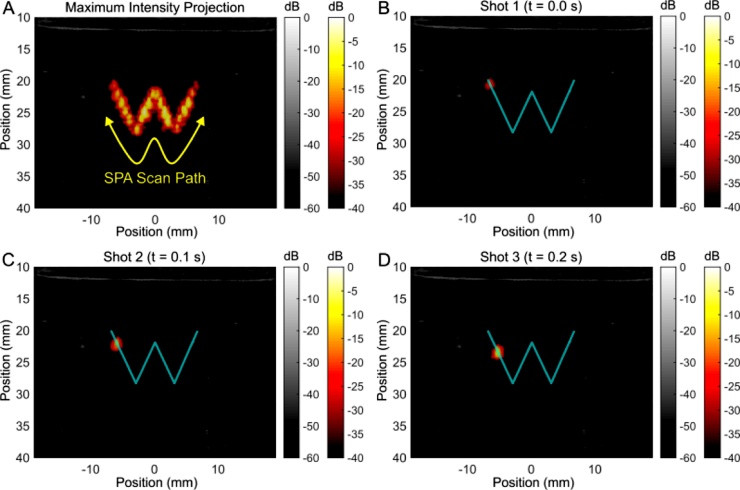
Demonstration of steering SPA activation along an arbitrary path to draw a ‘W’ (A). (B-D) sequential SPA activation events 100 ms apart show no signs of the previous activation event. An acoustic peak negative pressure of 1.74 MPa (MI = 0.76) was used with a surface optical fluence of 28 mJ/cm^2^. The local optical fluence over the path was estimated to vary from 2.0 mJ/cm^2^ to 4.8 mJ/cm^2^.

Electronically steered activation was demonstrated on the PVA gel loaded with PPy coated perfluoropentane droplets. The acoustic focus was steered with a constant f-number of 2 and synchronized with the laser flash at the appropriate time to activate droplets at the acoustic focus. The scanner was programmed to trace a ‘W’ shaped path (Fig. 3, supplemental media 1). The ultrasound (grayscale colormap) and SPA contrast images (hot colormap) were normalized according to the maximum signal acquired during acquisition. The sequence covered 24 discrete points in 2.4 s per pass. The sequence was repeated 4 times sequentially, with no delay, changing the scan direction with each pass. SPA images (displayed from 0 to -40 dB) were superimposed on ultrasound images (displayed from 0 to -60 dB). The estimated local laser fluence ranged from 4.8 mJ/cm^2^ at the top of the ‘W’ down to 2.0 mJ/cm^2^ at its bottom. The pressure field at the focus was near constant from shot to shot because of the constant f-number, with a maximum peak negative pressure of 1.74 MPa (MI = 0.76).

Immediately after each activation event, no ultrasound contrast was visible from one SPA frame to the next, or even in the following B-mode image acquired within 10 ms after the initial activation pulse (Figs. 1B–D). This suggests that activated bubbles producing SPA contrast are not stable and not long-lasting. Moreover, droplet activation could be repeated along the same path numerous times, strongly suggesting that agents are recondensing into a liquid droplet after activation (supplemental media 1). Because droplets immediately recondense after activation, the activation path can be arbitrarily defined with no effect on activation from shot to shot.

### 3.2 Irreversible SPA droplet activation

**Fig. 4:**
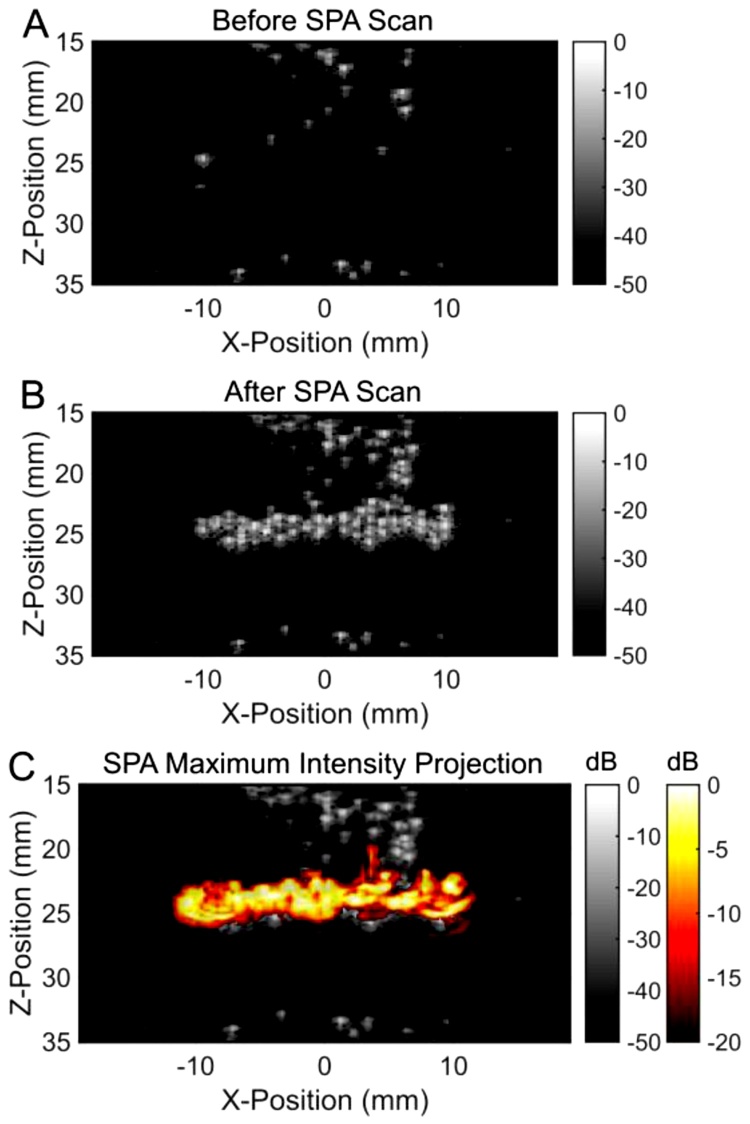
An example of irreversible SPA activation. B-mode ultrasound images (A) before and (B) after SPA activation of perfluorobutane (T_Boiling_ = −2 °C) droplets. (C) Maximum intensity projection of SPA contrast overlaid on the final ultrasound image. Due to the high volatility of perfluorobutane droplets, they did not return to the liquid phase after activation. This resulted in persisting ultrasound contrast seen in panel B.

An example of irreversible droplet activation was performed using a similar PVA gel loaded with PPy coated perfluorobutane nanodroplets (T_Boiling_ = −2 °C) (Fig. 4, supplemental media 2). The sequence covered 20 discrete points in 2.0 s, scanning from left to right. Images were normalized according to the peak signal acquired during acquisition and displayed from 0 to -20 dB for SPA images and 0 to -50 dB for ultrasound images. PPy coated perfluorobutane droplets were vaporized using acoustic pulses with a peak negative pressure of 1.74 MPa (MI = 0.76) and a local laser fluence of only 0.97 mJ/cm^2^. These more volatile droplets persisted in the gel as gaseous microbubbles, providing long lasting ultrasound contrast. Although providing excellent contrast at much lower activation thresholds than higher boiling point nanodroplets [29], persisting microbubbles can scatter or shadow acoustic and optical pulses. In applications where microbubbles (ultrasound scatterers) persist, such as histrotripsy, strategically prioritizing the firing sequence is needed to prevent interference from bubbles (e.g., shadowing effects or reflections), limiting uniform coverage of the treatment area. Although volatile nanodroplets with lower thresholds can be used at greater depths, higher boiling point nanodroplets can be repeatedly activated with arbitrary scan priority.

### 3.3 Independent lateral and axial SPA activation control

When droplets are activated using acoustic or optical vaporization alone, activation is localized by optical or acoustic focusing. It is very difficult to spatially localize optical activation at depth within the body because of diffusive optical scattering. Pure acoustic activation can be localized at depth using acoustic beam forming, similar to the approach in this study. The lateral full-width half max of the beam shape is directly related to the lateral extent of droplets activated. The axial extent, however, is determined primarily by the depth of field of the acoustic lens used to form the beam. In many cases at depth in tissue, the full-width half max of the axial beam determined by the depth of field is much larger than the lateral width. In any event, lateral and axial dimensions for acoustic activation are not independent because they are both related to the f-number of the acoustic lens. This is **not** the case for SPA activation of droplets.

**Fig. 5:**
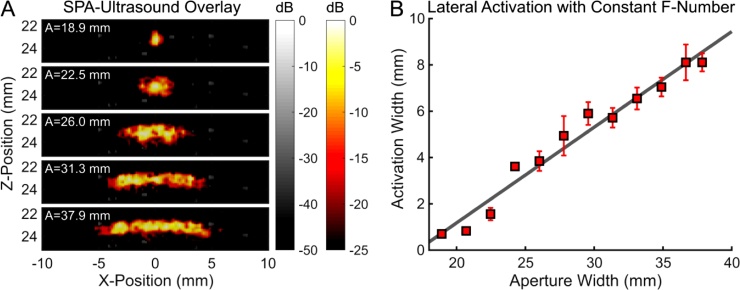
(A) The lateral width of SPA activation and image contrast can be modulated by changing the aperture width. (B) Using a constant laser flash delay and fixed f-number, changing the aperture size will proportionally change the lateral dimension of the activation volume.

The ability to modulate the lateral dimension of SPA activation was demonstrated using a fixed acoustic f-number (f = 1.3) and adjusting the acoustic transmit aperture width to move the focus of the ultrasound pulse closer to or further from the transducer. By flashing the laser when the acoustic wave hit a fixed depth of 23 mm, the lateral width of the propagating acoustic wave (and SPA contrast) varied depending on its relative distance from the acoustic focus (Fig. 5, supplemental media 3). The sequence used 12 unique aperture widths, opening and closing the aperture four times (48 total shots) over the course of 4.8 s. The images acquired were normalized according to the peak signal acquired during acquisition and displayed from 0 to -25 dB for the SPA contrast images and 0 to -50 dB for the ultrasound images.

Because of the change in both aperture size and depth relative to the focus of the transducer, the transmit amplitude was modulated to compensate for differences in pressure amplitude and to provide consistent contrast using a peak negative pressure of approximately 1.21 MPa (MI = 0.53). For the smallest acoustic apertures, the distance the acoustic wave propagated coincided with the acoustic focal spot when the laser was flashed. As the aperture increased, the lateral width of the propagating acoustic wave at the time the laser flashed increased due to an increasing distance between the acoustic focal length and the activation depth (Fig. 5A). As a result, the lateral activation width was proportional to size of the acoustic aperture (Fig. 5B).

The minimum lateral resolution for SPA activation is similar to that for purely linear acoustic activation methods, where the lateral resolution is defined by the minimum beam width at the time of activation. SPA activation differs from linear acoustic activation primarily along the axial direction, where SPA axial resolution is defined by the spatial distribution of sound when the laser is flashed. Because the laser pulse duration is short (∼ 5 ns) relative to the speed of sound, the laser flash only activates droplets during a time snapshot when the negative pressure combined with the local laser fluence is above the SPA threshold. This decouples the axial activation length from the characteristics of the acoustic lens.

**Fig. 6:**
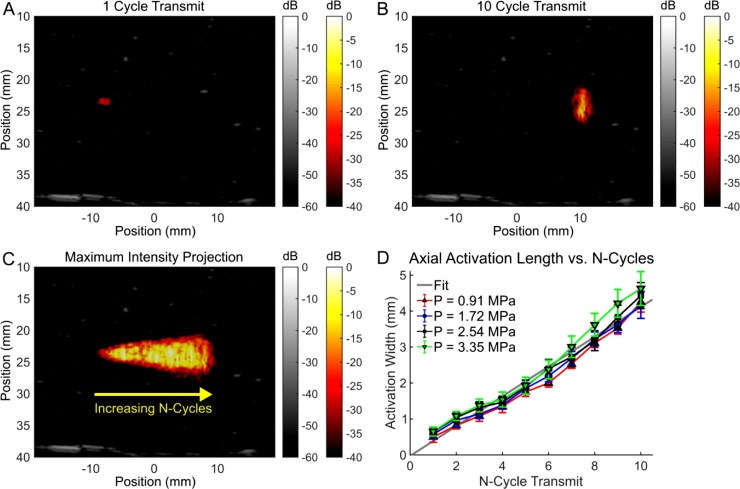
SPA activation with increasing N-transmit cycles from (A) 1 cycle up to (B) 10 cycles scanning linearly from left to right. (C) The composite frame for neighboring shots linearly increasing from 1 cycle to 10 cycles. (D) Axial activation length is approximately linearly proportional to the number of cycles used on transmit. An acoustic peak negative pressure of 1.74 MPa (MI = 0.76) was used with a surface optical fluence of 28 mJ/cm^2^. The estimated local laser fluence was estimated to vary from 4.8 mJ/cm^2^ down to 2.3 mJ/cm^2^ depending on the depth of activation.

The ability to vary the axial dimension of SPA activation was then demonstrated using a fixed acoustic aperture and f-number (f = 2) and only varying the pulse length (Fig. 6). Local laser fluences across the activation region are estimated to vary from 4.8 mJ/cm^2^ down to 2.3 mJ/cm^2^ depending on the activation position. An acoustic pressure of 1.74 MPa (MI = 0.76) was used for activation. In this sequence, SPA activation events were sequentially fired from left to right, where each activation event incrementally increased the number of cycles transmitted (Fig. 6 and supplemental media 4). The sequence covered 20 discrete points in 2.0 s, scanning from left to right. The images acquired were normalized according to the peak signal acquired during acquisition and displayed from 0 to -40 dB for SPA images and 0 to -60 dB for ultrasound images. Below the activation threshold, no droplet activation and contrast were observed. As long as SPA activation was above threshold, the axial activation length was nearly linearly proportional to the number of cycles transmitted and did not vary with pressure (Fig. 6D). Consequently, the axial SPA activation length can be easily varied from a single acoustic wavelength up to 10 wavelengths, while the lateral activation width remained unchanged (2.8 mm ± 0.3 mm). Independent control of lateral and axial dimensions is a very powerful tool for practical sequence design to cover complex geometries rapidly and efficiently.

### 3.4 Minimum SPA activation dimensions

It is possible to achieve half-wavelength SPA resolution because activation only occurs in the rarefaction phase of the US pulse. During single cycle SPA activation, each non-linear photoacoustic (${\text{N}\text{L}\text{P}\text{A}}_{+/-}=\text{P}\text{A}US_{+/-}-US_{+/-}$) component only activates a half wavelength of droplets. For SPA imaging with half-cycle resolution, the SPA sequence must be modified to $SPA_{\frac{1}{2}\;Cycle}\;=\left(PAUS_{1\;Cycle}-US_{1\;Cycle}\right)-PA$. The $\left(PAUS_{1\;Cycle}-US_{1\;Cycle}\right)$ component activates droplets and eliminates linear ultrasound scattering from the background signal, leaving linear PA signals from background light absorption mixed with signals from droplet activation. The final PA signal subtraction removes all linear PA single sources, such as blood, fat, etc., isolating the droplet activation signal and leaving a half cycle resolution SPA image.

**Fig. 7:**
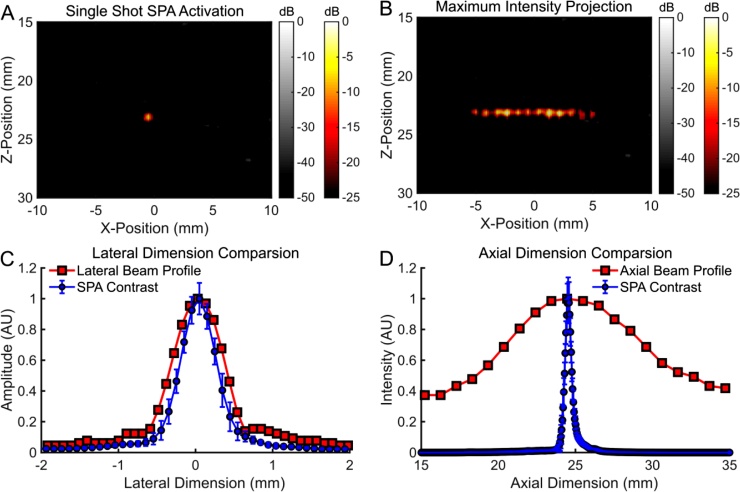
(A) A single frame versus (B) the maximum intensity projection of 12 shots along a horizontal line using a half cycle SPA activation sequence. (C) The lateral dimension of the SPA contrast observed corresponded very well with hydrophone measurements of the acoustic beam profile. (D) The axial dimension of activation width was significantly smaller than the acoustic beam profile determined by the lens depth of field.

This sequence was demonstrated by sequentially activating PPy coated perfluoropentane droplets loaded in a PVA gel along a linear path (Fig. 7, supplemental media 5). The scan sequence covered 12 discrete activation points in 1.2 s, repeating the scan four times. The images acquired were normalized according to the peak signal acquired during acquisition and displayed from 0 to -25 dB for SPA images and 0 to -50 dB for ultrasound images. A local laser fluence of 3.1 mJ/cm^2^ was used in combination with an f = 1.75 acoustic aperture outputting a peak negative pressure of 1.1 MPa (MI = 0.48). The lateral dimension of SPA contrast (FWHM = 0.54 mm ± 0.06 mm) corresponded very well with hydrophone measurements of the acoustic beam profile (FWHM = 0.63 mm) (Fig. 7C). This makes sense as the light is diffuse and, therefore, lateral activation should be limited to the acoustic diffraction limit. The axial activation length (FWHM = 0.47 mm ± 0.05 mm) was significantly smaller than the acoustic beam profile (FWHM = 10.6 mm) (Fig. 7D), which is determined primarily by the depth of field of the acoustic lens. Because only one acoustic cycle was transmitted coincident with a 5 ns laser flash, the axial dimension of the activation region should, indeed, be much smaller than the acoustic beam profile. However, the contrast observed along the axial dimension of the activation volume (0.47 mm ± 0.05 mm) is slightly larger than one acoustic wavelength (0.296 mm at 5.2 MHz). This minor discrepancy in the minimum axial activation resolution compared to one wavelength is most likely due to bubble ringing or broadband acoustic emission produced from the vaporization process.

## 4 Conclusions

SPA activation of phase-change contrast agents is highly spatially selective, far exceeding the localization capabilities of either pure optical or acoustic activation at depth within tissue. A local combination of light, sound, and agent is required to produce image contrast through droplet vaporization. In this study, highly localized droplet activation was demonstrated using an SPA sequence with focused acoustic beams. The lateral activation width was defined by the acoustic beam width at the time of laser excitation. However, the axial beam width was defined by the number of cycles used during transmit rather than the acoustic lens’ depth of field. Because the laser pulse was short (∼5 ns) relative to the propagation time of the acoustic wave, only droplets located in the rarefaction phases of the propagating acoustic field were vaporized.

Questions surrounding in vivo implementation of SPA imaging and therapy still remain. In its current state, SPA imaging is limited to 10 Hz because two laser shots are needed to reconstruct a single SPA image and the maximum pulse repetition rate from the laser used in this study is 20 Hz. As a result, PA motion artifacts between laser shots are likely, but can be improved in the future as faster lasers become available. However, even with the current system, motion artifact from US subtraction are minimal because US and PAUS frames are acquired about 100 μs apart. Heterogeneities in tissue optical properties greatly limit PA imaging depth and quantification. However, for SPA contrast and theranostics, the ability to dynamically compensate for low optical fluences by increasing acoustic pressure means that tissue heterogeneities will have a lesser effect [29].

The ability to smoothly transition from acoustic plane-waves to tightly focused beams to modulate lateral activation width, while independently controlling the axial activation length by transmitting N acoustic cycles, can help optimize molecular theranostic applications of phase-change agents. Like other molecular imaging approaches, PFC-based nanoemulsion agents can accumulate in disease sites through physiologic processes such as the EPR effect or by molecular targeting. A plane-wave activation sequence using low intensity plane-wave ultrasound and laser fluences can be used as a robust contrast-enhanced diagnostic tool to identify diseased tissues based on particle accumulation. Once the disease site has been identified, focused acoustic beams can be used for greater spatial selectivity and higher acoustic pressures, enabling safe targeted ablation of diseased tissue while minimizing collateral damage to surrounding healthy tissue. Because laser and acoustic energies are used synergistically to vaporize nanodroplets, near-diagnostic US and laser levels should be all that is required for efficient and highly localized ablation therapy. The ability to adapt a clinical US scanner and laser source for diagnostic and therapeutic modalities is an exciting possibility for nanodroplet-based molecular theranostics.

## Funding source

The research performed was primarily supported by the 10.13039/100000002National Institutes of Health under grant R01HL125339. Additional support was provided by National Institutes of Health grants R01EB016034, R01CA170734, R01EB009682, R01HL093140, R01DC010201, and R01EY026532.

## Declaration of Competing Interest

The authors declare that there are no conflicts of interest.
