# Supplementary material for: Spatially localized sono-photoacoutic activation of phase-change contrast agents
Source: Photoacoustics. 2020 Aug 3;20:100202. doi: 10.1016/j.pacs.2020.100202 (PMC7424230; doi:10.1016/j.pacs.2020.100202)
Supplement: Supplementary file 1 [file mmc1.pdf]

Supplemental Figures:

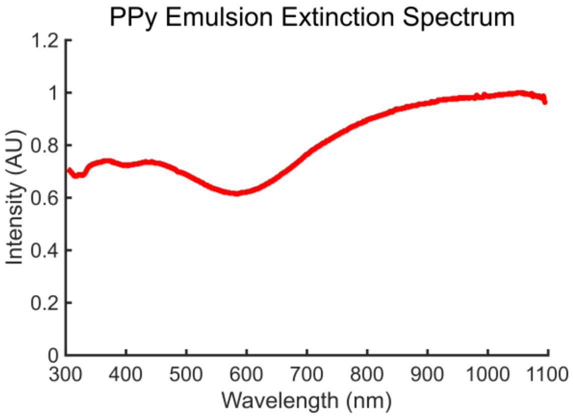

Supplemental figure 1: Representative optical extinction spectrum of polypyrrole (PPy) coated perfluorocarbon nanodroplets.

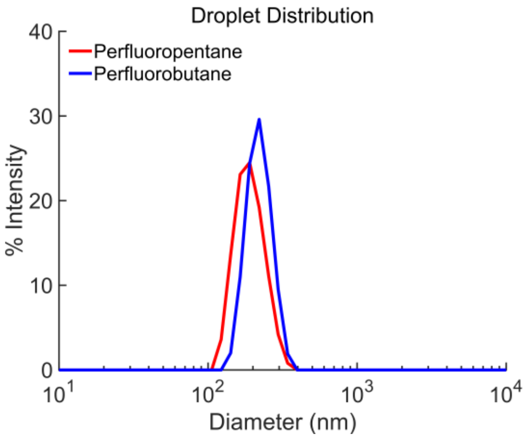

Supplemental figure 2: Droplet distributions used in PPy-coated perfluorocarbon droplet PVA gels.

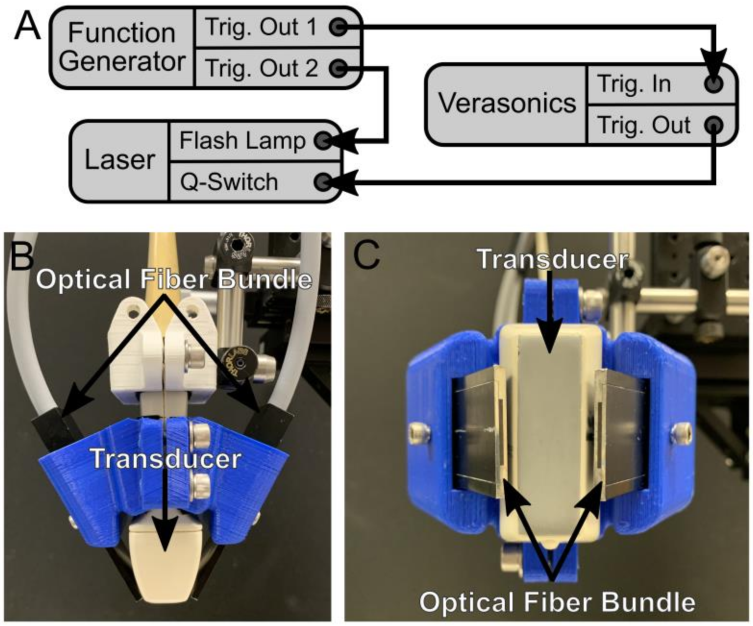

Supplemental figure 3: (A) Block diagram for triggering the SPA sequence. Trigger output 1 from the function generator is used to initiate the Verasonics sequence (shown in Figure 2). The delayed trigger output 2 from the function generator and the trigger output from the Verasonics are used to trigger the laser. (B) Side view and (C) front view of the optical fiber and ultrasound transducer arrangement.

Supplemental Media 1 – ‘Supplemental_Media_1_SPA_W.mp4’

Demonstration of steering SPA activation along an arbitrary path to draw a ‘W’. The left panel shows the shot-to-shot variation of SPA contrast with 3 frames of linear persistence. The right panel shows an integrated maximum intensity projection of SPA contrast throughout the scan. In both panels background ultrasound images are updated in real time. The images acquired were normalized according to the peak signal acquired during acquisition and displayed from 0 to -40 dB for SPA images (hot colormap) and -15 to -55 dB for ultrasound images (grayscale colormap). An acoustic peak negative pressure of 1.74 MPa (MI = 0.76) was used with a surface optical fluence of 28 mJ/cm^2. The local optical fluence over the path was estimated to vary from 2.0 mJ/cm^2 to 4.8 mJ/cm^2.

Supplemental Media 2 – ‘Supplemental_Media_2_Irreversible_SPA.mp4’

An example of irreversible SPA activation using perfluorobutane ($T_{Boiling}$ = -2°C) droplets. The upper panel shows the shot-to-shot variation of SPA contrast while the lower panel shows an integrated maximum intensity projection of SPA contrast throughout the scan. The images acquired were normalized according to the peak signal acquired during acquisition and displayed from 0 to -20 dB for SPA images (hot colormap) and 0 to -50 dB for ultrasound images (grayscale colormap). Due to the high volatility of perfluorobutane droplets, they did not return to the liquid phase after activation. This resulted in persisting ultrasound contrast seen in panel B. The PPy-coated perfluorobutane droplets were vaporized using acoustic pulses with a peak negative pressure of 1.74 MPa (MI = 0.76) and a local laser fluence of only 0.97 mJ/cm^2.

Supplemental Media 3 – ‘Supplemental_Media_3_Lateral_Control.mp4’

The lateral width of SPA activation and image contrast can be modulated by changing the aperture width. Acquired images were normalized according to the peak signal during acquisition and displayed from 0 to -25 dB for SPA images (hot colormap) and -15 to -55 dB for ultrasound images (grayscale colormap). Using a constant laser flash delay and fixed f-number,

changing the aperture size will proportionally change the lateral dimension of the activation volume. The activation width was modulated using a fixed f-number (f=1.3) and adjusting the acoustic transmit aperture width to move the focus of the ultrasound pulse closer to or further from the transducer. Because of the change in both aperture size and depth relative to the focus of the transducer, the transmit amplitude was modulated to compensate for differences in pressure amplitude and to provide consistent contrast using a peak negative pressure of approximately 1.21 MPa (MI = 0.53).

Supplemental Media 4 – 'Supplemental_Media_4_Axial_Control.mp4'

SPA activation with increasing N-transmit cycles from 1 cycle up to 10 cycles from left to right. The lower panel shows the shot-to-shot variation of SPA contrast while the upper panel shows an integrated maximum intensity projection of SPA contrast throughout the scan. Acquired images were normalized according to the peak signal during acquisition and displayed from 0 to -40 dB for SPA images (hot colormap) and -15 to -55 dB for ultrasound images (grayscale colormap). An acoustic peak negative pressure of 1.74 MPa (MI = 0.76) was used with a surface optical fluence of 28 mJ/cm^2. The local laser fluence was estimated to vary from 4.8 mJ/cm^2 down to 2.3 mJ/cm^2 depending on the depth of activation.

Supplemental Media 5 – 'Supplemental_Media_5_Minimum Resolution.mp4'

Scanning SPA activation with only half acoustic cycle activation. The upper panel shows the shot-to-shot variation of SPA contrast while the lower panel shows an integrated maximum intensity projection of SPA contrast throughout the scan. Acquired images were normalized according to the peak signal during acquisition and displayed from 0 to -20 dB for SPA contrast images (hot colormap) and -15 to -55 dB for ultrasound images (grayscale colormap). A local laser fluence of 3.1 mJ/cm^2 was used in combination with an f=1.75 acoustic aperture outputting a peak negative pressure of 1.1 MPa (MI = 0.48).
