# Supplementary figures and images for: Spatially localized sono-photoacoutic activation of phase-change contrast agents
Source: Photoacoustics. 2020 Aug 3;20:100202. doi: 10.1016/j.pacs.2020.100202 (PMC7424230; doi:10.1016/j.pacs.2020.100202)

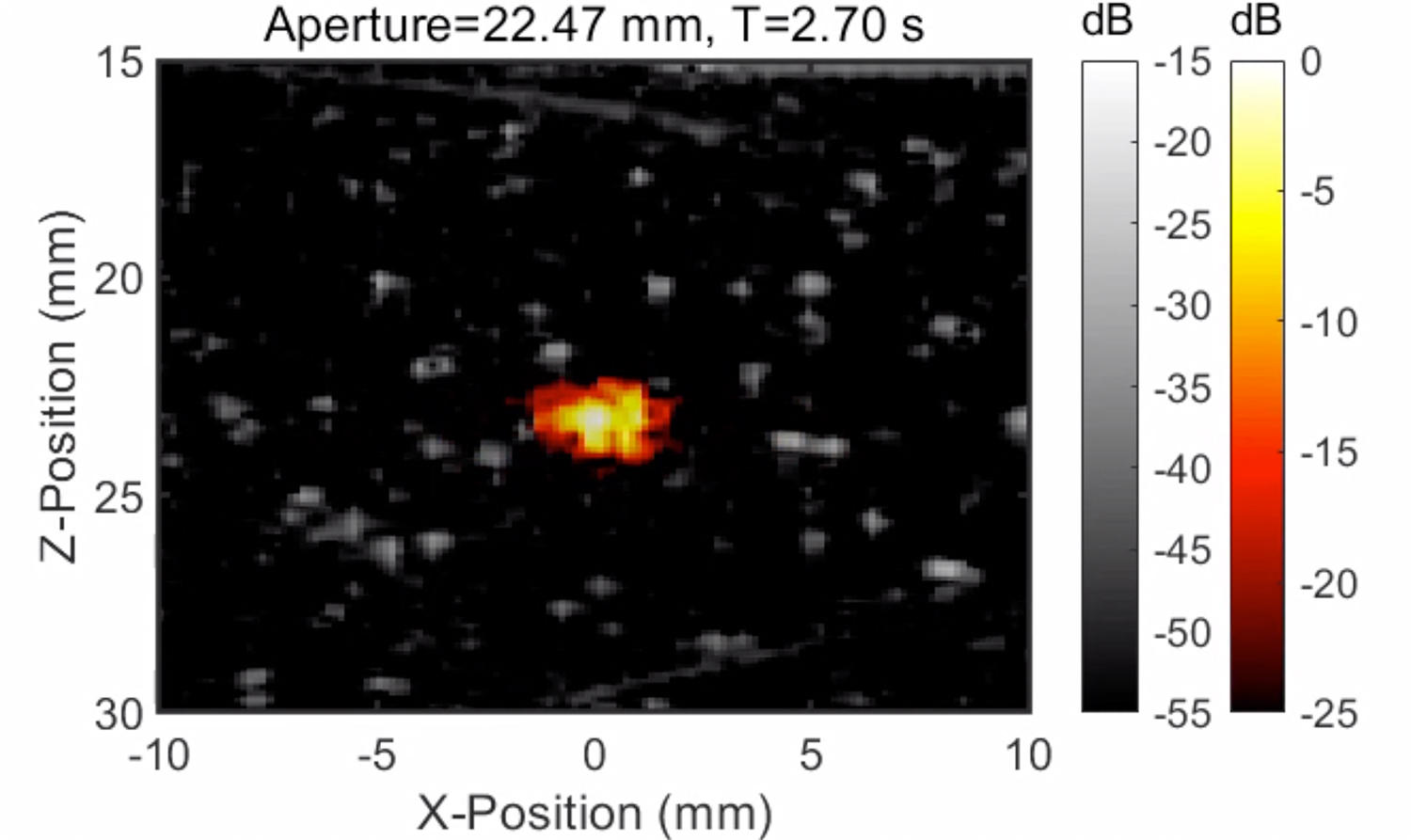

Supplement: Supplementary file 3 [file mmc3.zip › mmc3.jpg]

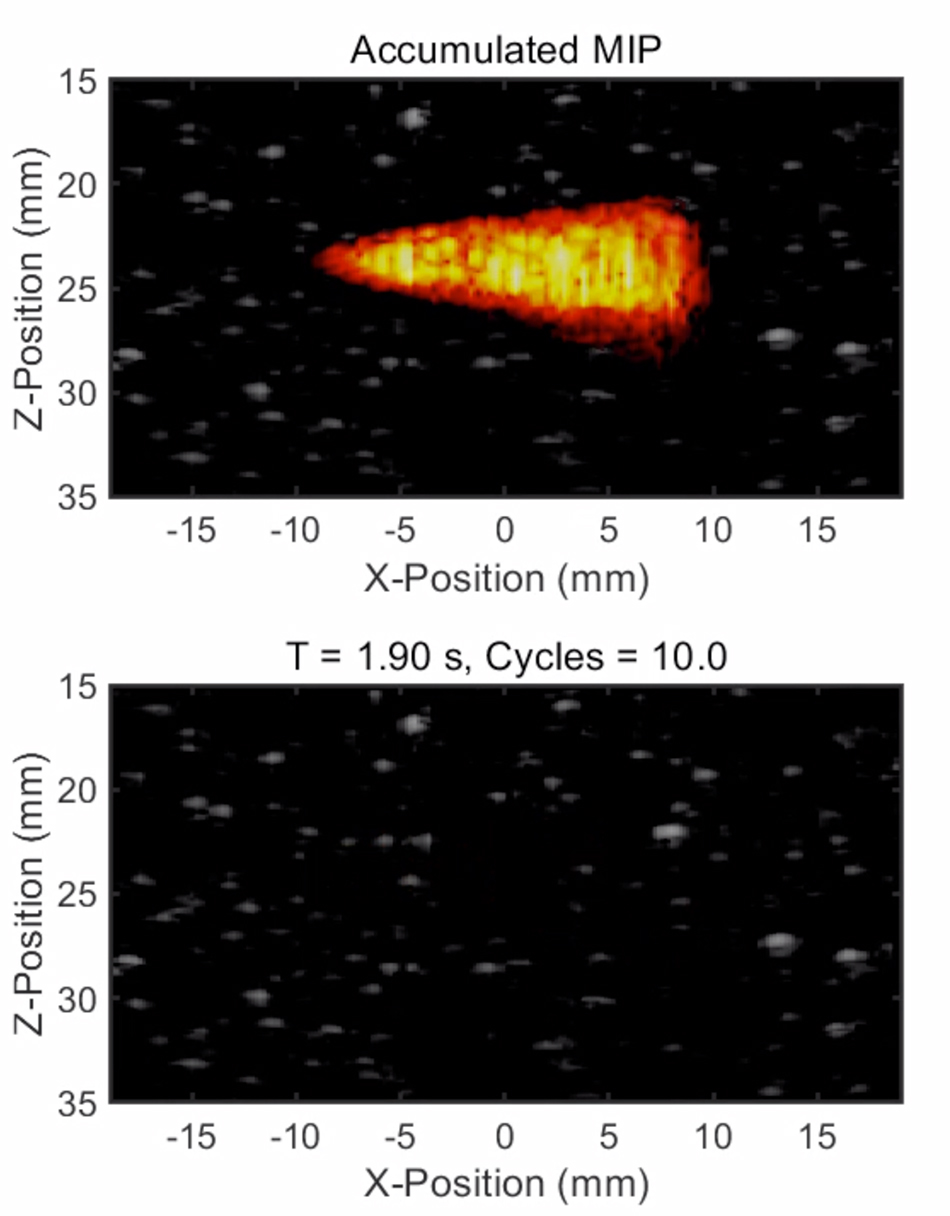

Supplement: Supplementary file 4 [file mmc4.zip › mmc4.jpg]

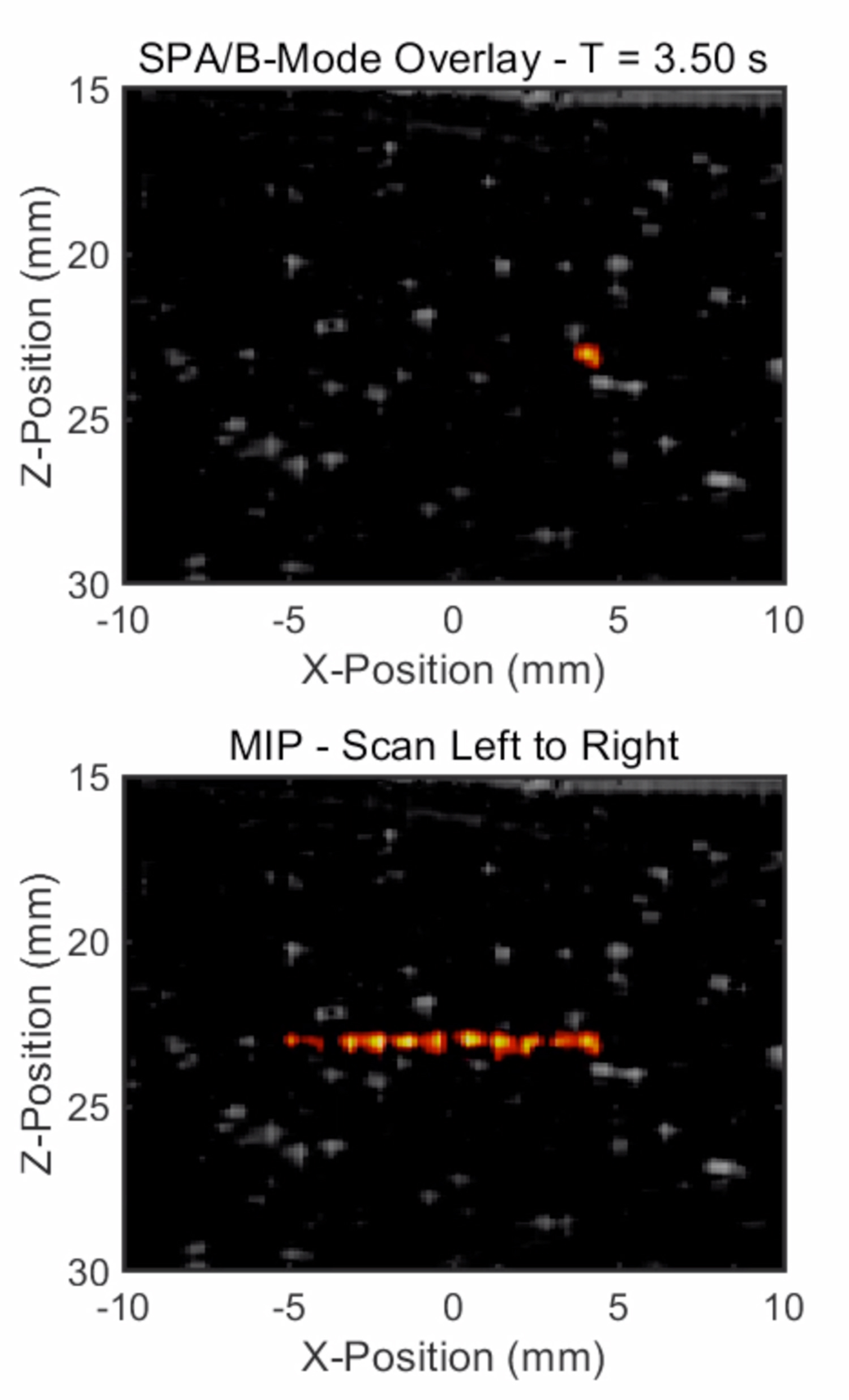

Supplement: Supplementary file 5 [file mmc5.zip › mmc5.jpg]

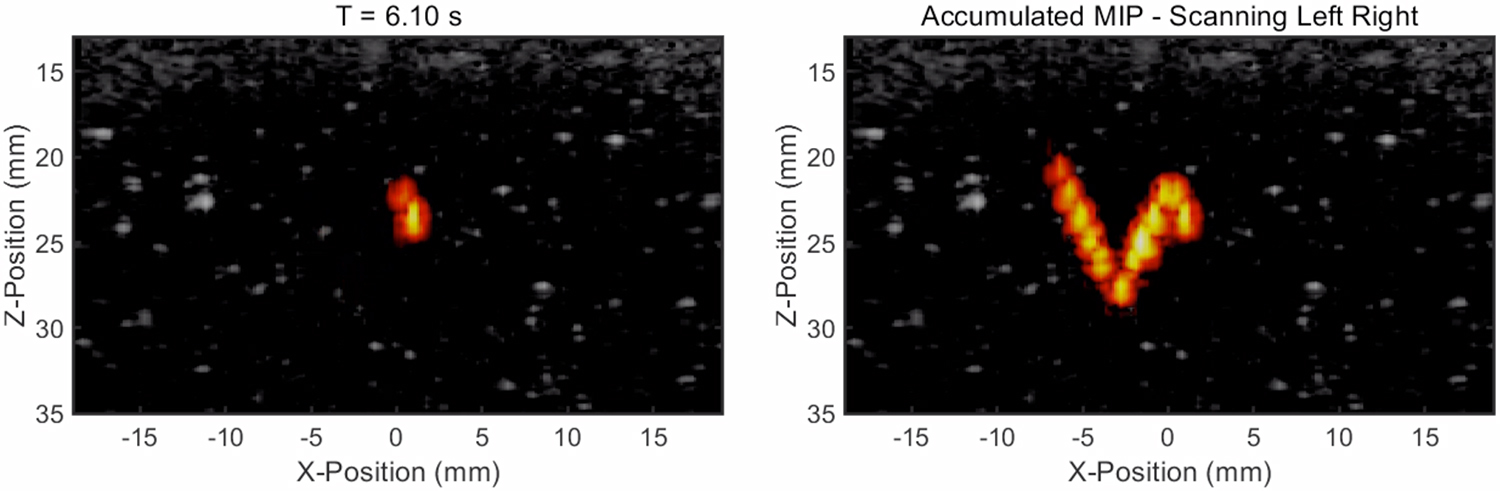

Supplement: Supplementary file 6 [file mmc6.zip › mmc6.jpg]
